# Utilization of Dental Services in a Field Practice Area in Mangalore, Karnataka

**DOI:** 10.4103/0970-0218.69278

**Published:** 2010-07

**Authors:** Sijan Poudyal, Ashwini Rao, Ramya Shenoy, Harsh Priya

**Affiliations:** Department of Community Dentistry, Manipal College of Dental Sciences, Manipal University, Mangalore, India

## Introduction

Oral health is the absence of disease and the optimal functioning of the mouth and its tissues, in a manner which helps to preserve high levels of self-esteem. Oral diseases such as dental caries, periodontitis and oral and pharyngeal cancers are a global health problem in both industrialized and especially in developing countries. In many developing countries, oral health care utilization is limited and teeth are often left untreated or extracted.

Utilization is the actual attendance by the members of the public at health care facilities to receive care. Utilization, which measures the number of visits per year or the number of people with at least one visit during the previous year,(1) serves as an important tool for oral health policy decision-making.(1,2)

The main objective of any health-care system is to maintain and improve health outcomes which depends on adequate knowledge of the way that the individuals use health services, and the factors predictive of this behavior.(3) Regular home oral care and a yearly dental check-up are the best means for caring teeth but in spite of the information on adequate dental care provided by the dental professionals and the mass media, many people fail to take these precautions.(4)

Barriers to seeking dental services as classified by the Federation Dentaire Internationale are related to the following:

- Individuals themselves (lack of perceived need and access, anxiety or fear and financial considerations),
- Dental profession (inappropriate manpower resources, uneven geographical distribution, training inappropriate to changing needs and demands and insufficient sensitivity to patient’s attitudes and needs), and
- Society (insufficient public support of attitudes conducive to health, inadequate oral health care facilities, inadequate oral health manpower planning and insufficient support for research).(5)

Utilization of dental services, however, is low and barriers to dental care have been of concern to those both within and outside the profession.(6)

## Aim

To determine the factors related to utilization of dental services in a field practice area in Mangalore, Karnataka.

## Materials and Methods

A house-to-house survey was conducted in the field practice area of Jeppinamogaru, Mangalore, Karnataka where dental services are provided for free of cost. The area consisted of 600 houses as stated in voter's list provided by the Gram Panchayat. A pilot study was conducted on 20 houses to test the feasibility of the study. Random sampling was done and 175 houses were selected. After obtaining consent, questionnaires were distributed to 195 adults above 18 years residing in the selected house to assess their dental utilization behavior. The questionnaires were collected after three days. A total of 182 completed questionnaires were received from 158 houses. Data were analyzed using SPSS version 11.5. Chi square and Fisher’s exact tests were used for comparison of categorical data. Level of statistical significance was set at *P*<0.05.

## Results

One hundred and eighty two completed questionnaires were used in the analysis. The percentage of participating female was 65.9 and male was 34.1. Mean age of the participants was 36.52±13.4 years (range 18-70) and 50% of them were of younger age group (18-34 years). Majority of them were Hindus 59.9%, followed by Muslims 34.6% and Christians 5.5%. Owing to low number of Christians and Muslims, the category of religion was divided into Hindus (59.9%) and non-Hindus (40.1%) for descriptive statistical purpose. Socio-economic status as determined by Kuppuswamy’s scale(7) showed 70.9% belonged to lower, 24.7% to middle and 4.4% to upper. A percentage of 28.6 (*n*=52) never visited a dentist, whereas 67% (*n*=122) visited a dentist only when they felt it was needed. Forty-four per cent (*n*=80) had dental diseases out of which 46.3% had not visited a dentist for that particular problem. The most common dental problem was toothache, followed by tooth decay, mobile teeth and bleeding gums [Table 1]. Dental visit experience for 41.22% (*n*=47) was very satisfactory. Highly reported reason for not visiting the dentist in the past was *“I haven't had any problems with my teeth”* followed by lack of time and fear of painful dental procedures [Table 2]. Fear was perceived by more females than males (*P*=0.03). Pattern of visiting a dentist was significantly different among Hindus and non-Hindus. More Hindus were very-satisfied with the past-dental visit than non-Hindus (*P*=0.04) and more non-Hindus were afraid during their past dental visit (*P*=0.007). Majority of them (75.8%) were aware of the fact that free preventive dental procedures were being provided at the center.

**Table 1:** Dental problems at the time of study

Dental problems	Frequency (%)
Toothache	41 (42.27)
Tooth decay	29 (29.90)
Mobile tooth	10 (10.30)
Bleeding gums	8 (8.24)
Ulcer in mouth	3 (3.09)
Any other	6 (6.2)

Total	97

**Table 2:** Reasons for not visiting the dentist in the past

Reasons	Frequency (%)
No problems in my teeth	91 (60.7)
No time	19 (12.7)
Fear of painful procedures	18 (12)
Dental diseases are not very serious	7 (4.7)
Other reasons	5 (3.3)
Dental diseases cannot be treated by anybody	3 (2)
Dental disease will recur	3 (2)
Long distance	2 (1.3)
Earlier unpleasant experiences	2 (1.3)

Total	150

## Discussion and Conclusion

Nearly 30% of the studied population had never visited a dentist although 44% of them had dental problems at the time of the study and majority of them were aware that free preventive dental procedures is being provided nearby. The highly reported tooth problem was toothache which being an emergency condition should logically have forced them to visit a dentist, but nearly half of the people having problems had not visited a dentist. This might indicate that either the people are using home remedies for toothache like clove oil, chewing tobacco or are taking medicines by themselves. Majority of them said that they visited a dentist only when they felt they needed it. A vast difference has been reported in other studies(5) between the normative need and the felt need of the people, where 75% of the subjects were assessed as requiring dental treatment, whereas only 22% of them perceived any need. The highly reported reasons for not visiting a dentist in this study was *“I haven't had any problems with my teeth”* which is similar to previous study.(4) Lack of time, a major barrier for dental visit,^6^ was the second most reported barrier in this study. Fear was reported more by females compared to males. This study did not show significant difference between the dental visit history among males and females; however, previous studies reported that females were more frequent dental visitors than males.(1,3) No significant association was demonstrated when socio-economic status, religion and age were compared with dental visit history, past dental experience and reasons for not visiting the dentist in the past.

Utilization of oral health care has long been used as an indicator of oral health related behavior. The highly reported reason for not visiting a dentist in this study was “I haven’t had any problems in my teeth” indicating the low felt need of the people in the area, which calls for improving their awareness and motivating them to use the services available to them so that they can lead a socially and economically productive life.

## References

[1] Manski RJ, Moeller JF, Maas WR (2001). Dental services, an analysis of utilization over 20 years. J Am Dent Assoc.

[2] Maserejian NN, Trachtenberg F, Link C, Tavares M (2008). Underutilization of dental care when it is freely available: A prospective study of the New England children’s amalgam trial. J Public Health Dent.

[3] Skaret E, Raadal M, Kvale G, Berg E (2003). Gender-based differences in factors related to non-utilization of dental care in young Norwegians. A longitudinal study. Eur J Oral Sci.

[4] Syrjälä AM, Knuuttila ML, Syrjälä LK (1992). Reasons preventing regular dental care. Community Dent Oral Epidemiol.

[5] Al-Shammari KF, Al-Ansari JM, Al-Khabbaz AK, Honkala S (2007). Barriers to seeking preventive dental care by Kuwaiti adults. Med Princ Pract.

[6] Tobias B, Smith JM (1987). Barriers to dental care, and associated oral status and treatment needs, in an elderly population living in sheltered accommodation in West Essex. Br Dent J.

[7] Kumar N, Shekhar C, Kumar P, Kundu AS (2007). Kuppuswamy’s Socio-economic Status Scale-Updating for 2007. Indian J Pediatr.

